# How service modularity can provide the flexibility to support person-centered care and shared decision-making

**DOI:** 10.1186/s12913-021-07267-6

**Published:** 2021-11-18

**Authors:** E. A. Bartels, B. R. Meijboom, L. M. W. Nahar-van Venrooij, E. de Vries

**Affiliations:** 1 Department of Management, Tilburg University, PO Box 90153, 5000LE Tilburg, The Netherlands; 2 Tranzo, Tilburg University, PO Box 90153, 5000LE Tilburg, The Netherlands; 3 Department of Marketing, Innovation and Organization, Ghent University, Tweekerkenstraat 2, 9000 Ghent, Belgium; 4 Jeroen Bosch Academy Research, Jeroen Bosch Ziekenhuis, PO Box 90153, B1.02.014, 5200ME ‘s-Hertogenbosch, The Netherlands

**Keywords:** Service modularity, Person-centered care, Shared decision-making

## Abstract

**Background:**

Today’s healthcare provision is facing several challenges, that cause the level of complexity to increase at a greater rate than the managerial capacity to effectively deal with it. One of these challenges is the demand for person-centered care in an approach that is tuned towards shared decision-making. Flexibility is needed to adequately respond to individual needs.

**Methods:**

We elaborate on the potential of service modularity as a foundation for person-centered care delivered in a shared decision-making context, and examine to what extent this can improve healthcare. We primarily focused on theory building. To support our effort and gain insight into how service modularity is currently discussed and applied in healthcare, we conducted a scoping review.

**Results:**

Descriptions of actual implementations of modularity in healthcare are rare. Nevertheless, applying a modular perspective can be beneficial to healthcare service improvement since those service modularity principles that are still missing can often be fulfilled relatively easily to improve healthcare practice. Service modularity offers a way towards flexible configuration of services, facilitating the composition of tailored service packages. Moreover, it can help to provide insight into the possibilities of care for both healthcare professionals and patients.

**Conclusions:**

We argue that applying a modular frame to healthcare services can contribute to individualized, holistic care provision and can benefit person-centered care. Furthermore, insight into the possibilities of care can help patients express their preferences, increasing their ability to actively participate in a shared decision-making process. Nevertheless, it remains essential that the healthcare professional actively collaborates with the patient in composing the care package, for which we propose a model. Altogether, we posit this can improve healthcare practice, especially for the people receiving care.

**Supplementary Information:**

The online version contains supplementary material available at 10.1186/s12913-021-07267-6.

## Background

Healthcare management increasingly faces a challenging problem: complexity in healthcare provision is increasing at a greater rate than the managerial capacity to effectively deal with it [1]. Healthcare provision is becoming more complex, due - among other things - to 1. more heterogeneous patient groups, e.g., because of comorbidities, 2., the increased complexity of care provision for one patient, e.g., because of the involvement of multiple healthcare providers, and 3., increased specialization of healthcare providers [2]. As a result, ‘one size’ no longer ‘fits all’; each person requires a tailored care package [1]. Furthermore, patient empowerment has increased over the years [1, 3]. People now demand active participation in the decision-making regarding their care, and attention to other aspects than just their biomedical situation. This requires a different attitude when interacting with patients as well as flexibility to adequately respond to the person’s preferences. We pose that service modularity, which is increasingly used in healthcare provision [4], can offer the flexibility needed to achieve this, but only when it is embedded in a person-centered approach that is tuned towards shared decision-making.

The concept of modularity has its origin in manufacturing and its application evolved from products to services [4]. *Service modularity* is based on the decomposition of the total service offering into multiple, largely independent parts, so-called modules. A module is “a relatively independent part of a system with a specific function and standardised interfaces, where the system can be, for example, a service, a service production process or an organisation or a network of organisations.” ([5] p47). These modules (e.g., consultations), in turn, each consist of one or more components (e.g., diagnostic tests) [5]. Interfaces (e.g., electronic health record reports) provide interaction between modules and between components, arrange how they fit together, and manage how they connect and interact within the service package [6, 7]. A modular service offering in healthcare facilitates to serve a diverse, heterogeneous group of patients with a tailored service package, even when multiple care providers – sometimes from multiple organizations – are involved. It offers the opportunity for healthcare providers and patients to mix-and-match the most suitable parts to fulfil that particular person’s needs and preferences, while the costs are expected to remain limited and under control [8].

*Person-centered care* is care focusing on the person rather than the disease, leading to care which is responsive towards the person’s individual needs and preferences [9]. Person-centered care is increasingly paid attention to in healthcare literature and practice. It is based on *patient*-centered care but “broadens and extends the perspective of patient-centered care by considering the whole life of the patient.” ([10] p10). Whereas patient-centered care aims for a *functional* life, person-centered care aims for a *meaningful* life [10]. To achieve person-centered care, an individualized goal-oriented care plan with a holistic focus should be composed, thus addressing medical, functional, and social needs [10, 11]. Research suggests that person-centered care can be delivered effectively and has the potential to benefit the person receiving care, e.g., through better health outcomes, increased patient satisfaction, greater enablement, and better understanding between healthcare professional and the patient on treatment plans [9, 10]. Although healthcare providers increasingly aim to practice person-centered care, they often do not succeed in this effort [9, 12].

To ensure care is truly responding to the needs and preferences of the person receiving it, mutual involvement and collaboration of that person and the healthcare professional is needed during care composition and delivery [13]. This collaborative process is referred to as *shared decision-making*. Shared decision-making aims to stimulate individuals to act independently and make their own free choices, by providing information and supporting the decision-making process [14]. Research has shown that shared decision-making results in knowledge gain by patients, more confidence in decisions, more active patient involvement, and the election of more conservative treatment options. Despite these positive effects, shared decision-making is often insufficiently applied in practice [15, 16].

To be able to *deliver* the person-centered care that was shaped in a process of shared decision-making, a care package tailored to that specific person has to be composed, thus addressing medical, functional, and social needs. This inherently requires a flexible configuration of services. Theoretically, service modularity offers a way to organize the service offering in a way that such unique service packages can be composed for each individual without increasing overall costs [8]. However, this theoretical potential is not yet fully explored, nor is it amply applied in healthcare practice. Therefore, we decided to elaborate on the potential of service modularity as a foundation for person-centered care delivered in a shared decision-making context, and to examine to what extent this can improve healthcare. (A more detailed description of the theory of service modularity, person-centered care, and shared decision making can be found in the Additional file 1.)

To summarize, in this paper we focus on the extent to which service modularity can and does serve as a foundation for delivering person-centered care in a shared decision-making context. With this effort, we aim to provide insight into how service modularity can benefit healthcare practice, and more specifically, can benefit the people receiving care.

## Service modularity in healthcare

To support our effort, we conducted a scoping review to identify papers addressing service modularity in healthcare. This section starts with an overview of the scoping review process. Following, insight is provided into how and to what extent service modularity is currently applied and recognized in healthcare in general. Subsequently, we describe the design principles and design choices associated when pursuing service modularity. Notably, we found that the service specification process – the process in which the care package is composed – is underexposed in the current service modularity literature. Thereafter, we provide our view on what this process should look like in order to assign a central and active position to the person receiving care. Finally, we discuss the expected effects of applying service modularity to facilitate person-centered care and shared decision-making in healthcare practice.

### Scoping review

**Fig. 1:**
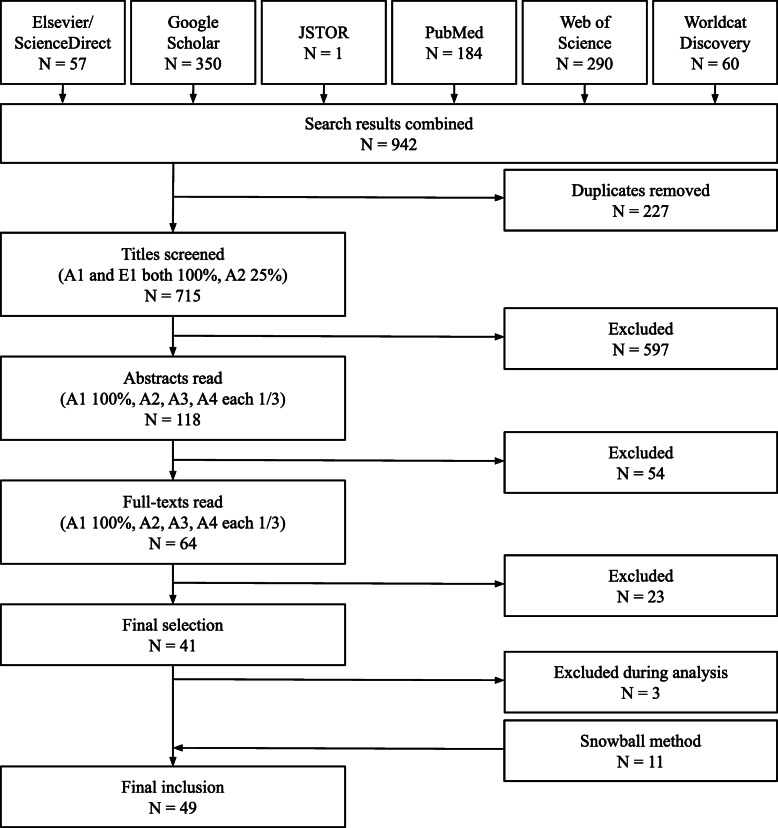
Flowchart Screening Process Scoping Review

We searched the databases of Elsevier/ScienceDirect, Google Scholar, JSTOR, PubMed, Web of Science & WorldCat Discovery systematically for articles published from 2000 until June 10th, 2020. These six databases were selected to cover a wide range of operations management as well as healthcare literature. We chose this time period since most articles concerning modularity in systems and services were published from 2000 onwards [17]. We used the search strategy [service] modularity AND health. All kinds of scientific publications (journal papers, conference papers, books, theses, proceedings, letter to the editor, etc.) written in English or Dutch, and discussing or applying service modularity in healthcare or when a healthcare setting was studied using a modular frame, were eligible for inclusion. The healthcare context studied in the paper needed to be directly related to patients; papers addressing healthcare logistics or strategic business approaches were excluded. After removing the duplicates, 715 unique papers remained for screening. The selection was performed in three phases: title screening, abstract reading, and full-text reading. Additionally, we scanned the reference lists of the included papers to identify additional eligible papers (‘snowball method’). The total screening process resulted in 49 papers eligible for inclusion (Fig. 1). We coded these papers in ATLAS.ti (version 8) using a thematic analysis based on the methodology proposed by Gioia, Corley, and Hamilton [18]. A more detailed description of the methodology can be found in Additional file 2.

### Modular healthcare services

We found that the number of published papers on service modularity in healthcare is limited. Papers discuss the subject either generally, or confined to a specific healthcare context, such as in-patient hospital care (hospitalization), out-patient hospital care, domestic/home care (hospital not involved), and mental care. Notably, most papers merely consider service modularity in healthcare conceptually or apply a modular perspective while addressing an already existing service. Descriptions of actual implementations of modularity in healthcare are rare [19]. This may be due to a lack of guidance on how non-modular care can be transformed into modular care in practice [19].

Some scholars mention that healthcare is in fact already a highly modularly organized sector (e.g., [20–22]). Others even argue that it is difficult to find examples of non-modular organized healthcare services [23]. Various healthcare organizations use a structure that enables them to combine a wide variety of independently functioning service parts in a fairly efficient manner into tailored care packages [21]. For example, Fransen et al. [6] studied chronic healthcare in a heterogeneous patient group using a modular frame, which resulted in the recognition of modules (e.g., consultation with physiotherapist, dietician), components (e.g., physical examination, blood test) and interfaces (e.g., multidisciplinary team meetings, electronic health record reports). Moreover, in care pathways and care protocols modules and components can be recognized in the various steps defined [24]. However, most healthcare providers are not aware of this. They do not know the service modularity literature, and do not apply a modular perspective when evaluating their services [22, 23].

Conversely, applying a modular perspective can be beneficial to healthcare service improvement since those service modularity principles that are still missing can often be fulfilled relatively easily to improve healthcare practice [20, 22]. The most common lack is that of well-defined broadly used interfaces [6, 20, 21, 23, 25]. Moreover, Peters et al. [25] found that little attention was paid to interfaces between the healthcare provider and the patient [6, 25] that support direct interaction (e.g., needs assessment, telephone consultation, patient portal) and indirect information exchange (e.g., information letter, consultation scheme) [26]. When striving for active participation in the decision-making process of the person receiving care, these interfaces between healthcare provider and patient are indispensable.

### Modular service design process

Some scholars address the process of designing a modular service provision [27, 28]. Three design principles are crucial for modular services offerings: specific function, relative independence, and standardized interfaces [2]. Each module should have a specific function that contributes to the overall service offering. Relative independence should exist between modules; the components within one module have strong interdependencies and are as little as possible connected to components in other modules. Modules should have standardized interfaces that allow for interaction and communication between them. These interfaces are important elements for making the modular service a functional whole [2, 21].

Broekhuis et al. [27] reformulated the three core design principles for modules into five design choices: number of decomposition layers (i.e., single- or multi-layered), decomposition orientation (i.e., outcome-, process- or actor-oriented [29]), degree of relative independence (i.e., interdependence between parts), degree of interface standardization (e.g., rule-based referral), and degree of within-module standardization (e.g., leaving room for professional autonomy) [27]. As addressed by Van der Laan [30] in her dissertation’s general discussion, especially the decomposition orientation can be relevant for person-centered care and shared decision-making. An outcome-orientated decomposition focuses on “what” services are delivered [28]. A process-orientated decomposition focuses on “how” the service is delivered. An actor-oriented decomposition focuses on “who” is involved in delivering the service [29]. An outcome-oriented decomposition is most likely to promote person-centered care and shared decision-making: due to the transparent display of the different options and corresponding benefits, the person can be involved to a greater extent in the composition of the care package [30]. The decomposition orientations are not mutually exclusive but can be combined, distributed over different decomposition layers [27].

We posit that a process-oriented first decomposition layer can provide guidance to the patient and the healthcare professionals involved. Many care processes comprise similar stages, which often proceed in a similar chronological order (e.g., diagnosis, treatment, aftercare). Therefore, most patients and healthcare professionals are familiar with these stages. A modular service offering, in which the first decomposition layer represents these familiar stages, supports the dialogue between the healthcare provider and the patient regarding the position and progress in the care process. Additionally, they can focus on the activities and possibilities per stage, which we expect can help to tailor information and structure the decision-making process. Altogether, a process-oriented first decomposition layer will contribute to well-coordinated care.

From the second decomposition layer, we expect an outcome-oriented decomposition is most favorable for person-centered care and shared decision-making. An outcome-oriented decomposition supports the composition of a goal-oriented care plan, which is one of the important elements for providing person-centered care [11]. To construct the outcome-oriented modules, we suggest asking the patients already receiving such care what their objectives are and how the care providers can best facilitate them in meeting these objectives. This information can be used to construct outcome-oriented modules and formulate the modules’ aims in accordance with peoples’ needs and wishes, which can cover both medical and non-medical needs. We expect this eases the communication between the patient and the healthcare professional, which supports shared decision-making: when composing the care package, the healthcare professional and patient together can select the modules and components which correspond best with the patient’s objectives.

Whether an actor-oriented decomposition is favorable for providing person-centered care is more ambiguous. Patients will not always know who can best facilitate them in meeting their needs and wishes. Their primary concern is often the outcome of the care they receive, rather than who provides this care. However, many patients want to be able to choose who (e.g., doctor, nurse practitioner) they consult with and who provides their care, based upon whom they trust and feel most comfortable with.

### Service specification process in practice

In the service modularity literature, customization and personalization are important concepts. Customization means tailoring the content of the service package [31]. In modular services, customization is achieved by mixing-and-matching modules and components to meet complex and diverse demands [20]. The process towards customization is referred to as the service specification process. Personalization in healthcare encompasses the adaptation of healthcare provider’s interpersonal behavior, for example by adapting the choice of words or way in which information is provided so that it suits a particular person’s preferences [31]. Case studies in healthcare have shown that personalization in care provision both complements and effectuates customization since patients feel more comfortable sharing their needs [31]. This allows healthcare professionals to optimally customize care.

When composing a modular care package during active care provision, customization and personalization are practiced simultaneously. Once care provision is ongoing, customization only takes place when the patient’s needs or preferences change, and the care package needs adaptation. Personalization remains important continuously [31], to enable the right atmosphere for continuous patient involvement. This is in line with the principles of person-centered care and shared decision-making. De Blok et al. [8] are one of the few scholars addressing this service specification process, in the context of long-term care provision. They recognized three phases of customization: a-priori specification, on-the-job adjustments, and ongoing care provision. The a-priori specification takes place before the care provision starts. A package of relatively standard care components is roughly defined and can be labelled as the provisional package. The person who is going to receive care often has a quite passive role in this phase. In the on-the-job phase, the components included in the package can be adapted and refined, taking the person’s specific characteristics and preferences into account. The service specification process as specified by De Blok et al. [8] is especially suitable for care delivery that can be relatively easily adapted over time. Depending on the healthcare context and possibility to adapt the care package over time, more emphasis can be put on, for example, the a-priori specification, allowing for more involvement of the person receiving care earlier in the process [8].

**Fig. 2:**
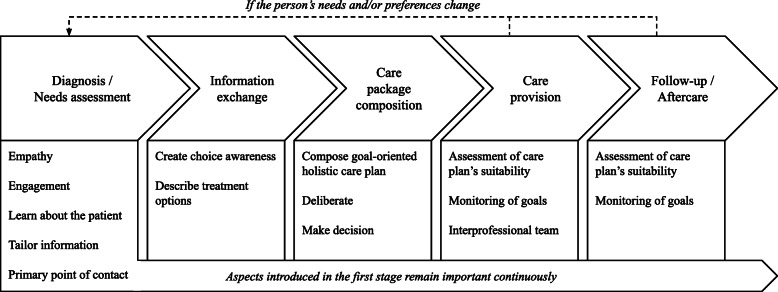
Person-Centered Service Specification Process in a Shared Decision-Making Context

We argue that, in order to assign the person receiving care a central position, factors and aspects related to person-centered care and shared decision-making should be explicitly integrated in a comprehensive model for the service specification process (Fig. 2). Comparing our model to the three phases distinguished by De Blok et al. [8], the a-priori specification is comparable with the diagnosis or needs assessment in our model. The on-the-job adjustments are comparable with the information exchange and the care package composition in our model; the person’s specific characteristics and preferences are taken into account to refine the initial care package. The ongoing care provision is similar to the care provision in our model. Unlike the service specification process defined by De Blok et al. [8], our model expanded the scope of the service specification model by adding the follow-up or aftercare as a stage. Personalization aspects are important regardless of the stage in the service specification process.

Following our model, the service specification process will start with diagnosing the person’s problem(s) and performing an initial needs assessment. Next, the healthcare professional and the patient will exchange information, among other things to create choice awareness and to describe the treatment options. After that, the actual goal-oriented holistic care plan will be composed, in a collaboration between the healthcare professional and the patient with room for deliberation and negotiation. In this stage, the decisions regarding care provision are made explicit and the person receiving care has the authority over these decisions. Once care provision starts, care is provided by an interprofessional team which the person receiving care is part of. Regular assessment of the care plan’s suitability is needed, and it should be monitored whether goals are achieved. Changes can still be made to the care package when needed or preferred by the person. This will result in repeating the needs assessment, information exchange, and care package composition. The care provision does not necessarily stop when changes need to be made but will continue as much as possible. After sufficient care has been provided, the follow-up or aftercare process will continue as long as needed. During this stage, it is possible that the person’s needs or preferences change (e.g., due to recurrence of the disease or changes in the person’s social life). Therefore, the care plan’s suitability should still be assessed regularly, and goals should continue to be monitored. This can result in going back to the diagnosis / needs assessment stage and going through all stages again.

The limited number of studies that pay attention to the service specification process (e.g., [21, 32, 33]) have shown that in practice, the professionals often combine the available treatment components without explicitly involving their patient to compose a service package. This also depends on the type of disease and necessary care. For instance, in oncology the treatment content is defined mainly according to cancer size, type of cancer cells, malignity, and presence of metastases [33]. Although patients’ lack of in-depth knowledge on the sometimes highly specialized treatment contents [8, 32] may result in them wanting to leave the responsibility with the healthcare professional, this does not mean it is impossible to compose the modular service package in a shared decision-making context. Doing this will increase the patient’s trust and wellbeing as well as compliance with the proposed treatment [10].

### Modular service potential for person-centered care in a shared decision-making context

So far, we showed how service modularity is currently applied and recognized in healthcare and what the service specification process should look like. In this section we explain the hypothesized potential of service modularity in supporting person-centered care and shared decision-making in healthcare, taking into account the point of view of the person receiving care. Moreover, we explain *why* we expect those effects. We chose to distinguish between effects directly involving the person receiving care and effects within the organization, thus indirectly affecting the person.

We argue the modules specified during service design in collaboration with patients should cover a broad range of services, thus addressing medical, functional, and social needs; this will promote a holistic view during the entire service provision. For example, when modules are included covering mental support, we expect the patient will more likely express his/her mental needs to the healthcare professional. The designed service should be presented in a clear, easy to comprehend, modular overview, giving patients information on what is possible, and enabling them to express their preferences. This in turn eases the communication between patient and healthcare professional, and as such, shared decision-making. Consequently, care can be better tailored to the person, increasing person-centered care.

**Fig. 3:**
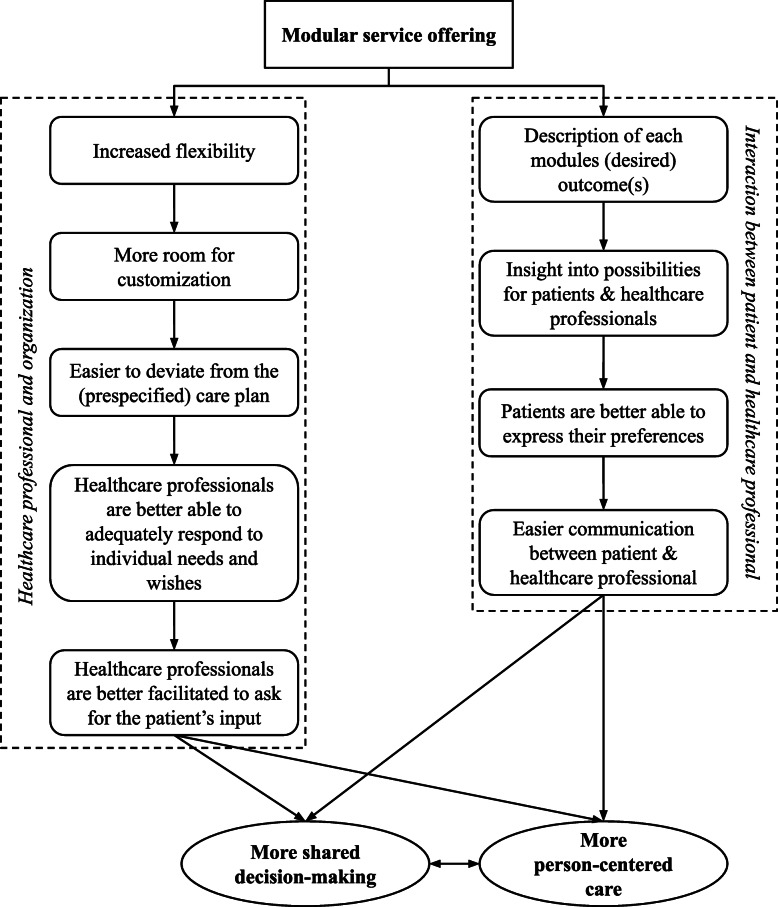
How a Modular Service Offering leads to more Person-Centered Care and Shared Decision-Making

A modular service offering increases the flexibility of care, and the ability to customize care packages. Consequently, it is easier to deviate from a prespecified care plan throughout the entire care process; modules and components can easily be replaced, modified, added or removed. The healthcare professionals are thus better able to continuously and adequately respond to the person’s needs and wishes. We argue this also results in healthcare professionals being more inclined to ask for the patient’s input, because they can respond to it. Altogether, this will result in more person-centered care, in which the person has a substantial position in the decision-making process (Fig. 3).

## Practice implications

As discussed above, we posit that a modular service offering in healthcare can contribute to individualized, holistic care provision, by offering the flexibility needed to achieve this. However, implementing the theoretical concept of service modularity in practice will not automatically lead to that result. In order to succeed, explicit attention should be paid to placing the person in the center of care, and to support him/her to take an active position in the process and to take part in the decisions to be made. Altogether, we posit that this way of improving the healthcare process enhances job satisfaction and quality of life for the people providing and receiving care, respectively.

It is worth noting that since healthcare services are often highly modular in nature, applying modular service design should not be seen as drastically changing current systems but rather changing the way of thinking, thereby recognizing the flexibility and possibilities for improvement already present under the hood. Describing existing healthcare practices in modular terms encourages healthcare professionals to reflect on both overlap in and completeness of care provision. It can help them to identify whether sufficient attention is paid to ‘the person behind the patient’ and to truly including the patient in a shared decision-making process, as well as to what extent care can be tailored to each person, already now, or in the future after some adjustments in the service specification process have been made.

Currently, healthcare practice often makes use of standardized care packages or pathways, which are efficient but may be rather rigid, and therefore, restrain individualized, holistic care provision. We do not suggest to fully eliminate these kinds of standardizations; especially in care provision that has great overlap between patients’ care processes these can be quite useful and can contribute to well-coordinated care. However, we encourage healthcare professionals to be open-minded towards possible deviations, in line with the patient’s needs and preferences. Not all patients prefer the longest life possible at whatever means; many value the quality of their life to an equal or even greater proportion [9]. We posit a modular set-up can support this way of thinking; when care is considered as a chain of separate parts (i.e., the modules) rather than as an integrated entity, the healthcare professional will more easily recognize opportunities for adjustment of these separate parts or addition of extra parts on the one hand and will realize more easily there is always a choice, even in the case of life-threatening conditions, on the other hand. This is essential for *holistic* care provision since this requires attention is paid to the person’s non-medical needs as well. Thus, the modular service offering should be composed based on a holistic approach; merely medical modules focusing on physical health (such as physical examination, blood test) will not result in care provision addressing the person as a whole. Since person-centered care aims for a meaningful life, the modules’ content should also cover daily functioning and mental well-being as well as time dedicated to assessing the patient’s viewpoint on life and care. Altogether, a person-centered modular service offering may consist of prespecified care packages – which can be altered if needed – addressing medical needs which are similar in most patients, as well as modules covering a variety of non-medical needs which often are variable in different persons.

Subsequently, even when the modular service offering is designed with a holistic approach, the process in which the care package is composed should be addressed adequately to successfully provide holistic care. An overview of the modules can be created to support insight into possibilities, choices and expected outcomes, which supports both healthcare professionals and patients during their conversations. The healthcare professional plays an important role in supporting the active involvement of the patient in the composition of the care package. It is indispensable that the healthcare professional adapts his/her interpersonal behavior to each individual patient, to make that patient feel at ease. Once a patient has been diagnosed, the healthcare professional should make the person aware of the medical and non-medical possibilities of care and choices that he/she can make. If the person is not aware non-medical support can be organized by the healthcare organization, we expect he/she will less likely address those needs in a conversation with the healthcare professional. As a result, the healthcare professional cannot address them in the care package. Additionally, the healthcare professional should express the expected outcomes of the different possibilities, not only with regards to physical but also non-physical outcomes. The healthcare professional should invite the patient to ask questions and to actively participate in the decision-making. Ultimately, this should lead to a well-tailored care package, composed by the healthcare professional and patient in collaboration. Once the actual care provision has started, the healthcare professional should regularly verify with the patient whether the care package still meets the patient’s needs and wishes. If needed, the content of the care package can be altered accordingly and thus remain tailored continuously.

We conclude that more practice-oriented research should be conducted, in which the model (Fig. 2) can be used as a conceptual ground for an empirical study. By understanding how service modularity can provide the flexibility to support person-centered care and shared decision-making, this paper provides a bridge between service modularity on the one hand, and person-centered care and shared decision-making on the other hand.

## Supplementary Information

**Additional file 1.** Theoretical concepts. More detailed explanation of service modularity, person-centered care, and shared decision-making.

**Additional file 2.** Methodology of scoping review. More detailed explanation of the methodology used for the scoping review.

## Data Availability

The dataset(s) (i.e., list of included articles) supporting the conclusions of this article is included within the article (and its additional file(s)).
