# Correction: How service modularity can provide the flexibility to support person-centered care and shared decision-making

**DOI:** 10.1186/s12913-022-08161-5

**Published:** 2022-06-23

**Authors:** E. A. Bartels, B. R. Meijboom, L. M. W. Nahar-van Venrooij, E. de Vries

**Affiliations:** 1 Department of Management, Tilburg University, PO Box 90153, 5000LE Tilburg, The Netherlands; 2 Tranzo, Tilburg University, PO Box 90153, 5000LE Tilburg, The Netherlands; 3 Department of Marketing, Innovation and Organization, Ghent University, Tweekerkenstraat 2, 9000 Ghent, Belgium; 4 Jeroen Bosch Academy Research, Jeroen Bosch Ziekenhuis, PO Box 90153, B1.02.014, 5200ME ‘s-Hertogenbosch, The Netherlands

**Correction: BMC Health Serv Res 21, 1245 (2021)**

**https://doi.org/10.1186/s12913-021-07267-6**

Following publication of the original article [1], the authors identified an error in one of the author names. The given names and family names of the third author were incorrectly tagged in the article metadata, resulting in an incorrect presentation of the author name on external indexing platforms.

The incorrect tagging of the author name is: L. M. W. Nahar-van (given names) Venrooij (family name).

The correct tagging of the author name is: L. M. W. (given names) Nahar-van Venrooij (family names).

The original article [1] has been corrected.

## References

[1] Bartels (2021). How service modularity can provide the flexibility to support person-centered care and shared decision-making. BMC Health Serv Res.

