# Supplementary material for: How service modularity can provide the flexibility to support person-centered care and shared decision-making
Source: BMC Health Serv Res. 2021 Nov 18;21:1245. doi: 10.1186/s12913-021-07267-6 (PMC8600923; doi:10.1186/s12913-021-07267-6)
Supplement: Supplementary file 1 — Additional file 1. Theoretical concepts. More detailed explanation of service modularity, person-centered care, and shared decision-making. [file 12913_2021_7267_MOESM1_ESM.docx]

# Additional file 1. Theoretical concepts.

In this additional file, we separately discuss the concepts of service modularity, person-centered care, and shared decision-making in more detail. First, we explain service modularity and its origin, definitions, and the role of the interaction between the service provider and customer. Second, we explain person-centered care and essential factors to establish person-centered care in practice. Third, we explain shared decision-making, its elements, and its barriers and facilitators.

## Service modularity

In 1962, Simon stated that he viewed complex systems as “one made up of a large number of parts that interact in a nonsimple way” [1 p468]. This is considered to be the foundation for modularity [2]. Originally, modularity was introduced in manufacturing, thus focusing on products [3]. However, since the turn of the millennium, researchers increasingly paid attention to the application of modularity to services. An important difference between manufacturing and service provision is the role of human behavior [4]. Whereas in manufacturing producer and customer act mostly separated, close interaction occurs between them in service provision.

Even though all modularity literature has the same origin, a variety of definitions, measures, and applications of the modularity concept exist in the literature [2]. We follow the definition posed by Rajahonka, Bask, and Lipponen [5], who define a service module as “a relatively independent part of a system with a specific function and standardised interfaces, where the system can be, for example, a service, a service production process or an organisation or a network of organisations.” [5 p47]. Modular services can be decomposed into these service modules, which, in turn, each consist of one or more service components [5]. An example of a module in healthcare is ‘consultation’, which consists of components such as ‘physical examination’ and ‘blood test’ [6]. Three design principles are crucial for modular services offerings: specific function, relative independence, and standardized interfaces [7]. Each module should have a specific function that contributes to the overall service offering. Relative independence should exist between modules; the components within one module have strong interdependencies and are as little as possible connected to components in other modules. Modules should have standardized interfaces that allow for interaction and communication between them. These interfaces are important elements for making the modular service a functional whole [7, 8]. They provide interaction between modules and between components, arrange how they fit together, and manage how they connect and interact within the service package [6, 9]. A distinction can be made between interfaces linking content with one another (e.g., protocols) and interfaces linking service providers (people) with one another (e.g., multidisciplinary team meetings) [7]. Interfaces linking components support the mixing-and-matching of a service offering, interfaces linking service providers facilitate the coordination of work between these providers. Remarkably, little attention has been paid so far to the interfaces between the service provider (the healthcare professional) and the customer (the patient) when discussing a healthcare context [10].

The decomposition of a service into modules and components connected by interfaces provides the opportunity for tailoring service packages, which is referred to as customization. Customization is the configuration of products and services that meet the person’s individual needs [4]. In modular services, customization is achieved by mixing-and-matching modules and components to meet complex and diverse demands [11]. This process towards customization is referred to as the service specification process and should entail collaboration between the service provider and the customer (i.e., the healthcare professional and the care receiver, respectively, in a healthcare context). Personalization in healthcare encompasses the adaptation of healthcare provider’s interpersonal behavior, for example by adapting the choice of words or way in which information is provided so that it suits a particular person’s preferences [4]. Due to the close interaction between the healthcare provider and the care receiver the concept of personalization plays an important role in service modularity in healthcare. Case studies in healthcare have shown that personalization in care provision both complements and effectuates customization since patients feel more comfortable sharing their needs [4]. This allows healthcare professionals to optimally customize care. Therefore, personalization – the adaptation of interpersonal behavior – and customization – tailoring the content – are inherently connected in service provision.

## Person-centered care

In *person*-centered care, the care composition and provision are focused on the person rather than the disease [12]. It is based on *patient*-centered care, which can be defined as “care that is respectful of and responsive to individual patient preferences, needs, and values” and that ensures “that patient values guide all clinical decisions.” [13 p780]. In addition to that, person-centered care “broadens and extends the perspective of patient-centered care by considering the whole life of the patient.” [14 p10]. To deliver person- instead of patient-centered care, it is even more important to shift from the patient as a passive target of the healthcare provision to the patient as a person playing an active role in his/her care and decision-making to the extent he/she desires [14, 15]. Besides, there is another important difference between patient- and person-centered care. Whereas patient-centered care aims for a functional life, person-centered care aims for a meaningful life [14]. Consequently, person-centered care places the person with his/her context, family, history, future plans, and individual strengths and weaknesses at the center even more than patient-centered care, and is responsive towards the person’s individual needs and preferences [13, 14].

To achieve person-centered care, it is important to know what the persons receiving care value in healthcare; this can relate to different aspects [16]. First of all, the person should be seen as an individual. An individualized goal-oriented care plan with a holistic focus should be composed, thus addressing medical, functional, and social needs [14, 15]. Besides, the way the healthcare professional behaves plays an important role in the practice of person-centered care [16]. The professional should empathize with the person's thoughts and experience and should have respect for his/her values, beliefs, and choices [14]. To facilitate this, attention must be paid to the notion of person-centered care in their education and training [15, 17]. Education and training should cover the understanding of and commitment to providing person-centered care and communicating in a person-centered care way [17]. Traditional approaches of healthcare provision, e.g., with standardized prescribing and in which the healthcare professional acts as the principal decision-maker should be abandoned. Also, in the interaction between the person receiving care and the healthcare professional [16], the professional should be engaged, meaning to be present and committed during encounters [14]. This builds trust and helps to build a relationship between the person and the professional. In turn, trust and a relationship positively affect two-way communication [14].

Since a person’s needs and preferences can change over time, it is important in chronic care to regularly monitor whether goals stated in the care plan are achieved and whether the care plan is still suitable [15]. To be able to continuously answer a person's needs and preferences, care must often be supported by an interprofessional team of which the person receiving care is part. The composition of this team must be adaptable to respond to changes in the care plan. One healthcare professional should serve as a primary point of contact within this team, managing the communication and information sharing between the person receiving care and the other professional team members [15].

In addition to the valuable factors visible from the patient’s point of view, also other factors are crucial to support adequate provision of person-centered care. In the interaction among the healthcare professionals, information relevant to person-centered care, e.g., the person's goal-oriented, holistic care plan, should be well-documented and accessible to all team members [15, 17]. Nowadays, the electronic health record can be used for this. Moreover, this primary point of contact coordinates the interprofessional collaboration [15]. Finally, supportive organizational conditions are vital. Healthcare professionals must have the ability to work flexibly and use different strategies to answer a person's needs and preferences [17]. Their workload should not be too high, enough time should be available to be able to deliver person-centered care. Also, when evaluating care, not only process measures (e.g., number of surgeries performed) should be taken into account, but also outcome measures, including patient experiences, to be able to include person-centered care aspects in the evaluation [15].

## Shared decision-making

To be responsive towards a person's preferences, needs, and values and to let these values guide all decisions, that person has to be actively engaged in making decisions regarding his/her care [13]. This can be achieved by shared decision-making. Shared decision-making is “an approach where clinicians and patients share the best available evidence when faced with the task of making decisions, and where patients are supported to consider options, to achieve informed preferences” [18 p1361]. Several scholars developed models to support the use of shared decision-making in practice. Bomhof-Roordink, Gärtner, Stiggelbout, and Pieterse [19] conducted a systematic literature review on the currently available shared decision-making models in the literature. The most frequently mentioned aspects of these shared decision-making models are: 1. describe treatment options, their feasibility, benefits and risks, and evidence of all options available; 2. explicitly make the decision, document it and revisit it when needed; 3. patient preferences regarding concerns, goals of care, and values; 4. tailor information, using clear language, and check whether the patient understands the information; 5. deliberate and negotiate; 6. create choice awareness by positioning the options in an open, non-directive manner, and by making the need for a decision explicit; 7. learn about the patient by checking or clarifying the healthcare professional’s understanding of the patient.

Elwyn et al. [18] developed a three-step framework to implement this in clinical practice. The first step is the "choice talk", in which the physician must make the patient aware there are various options and choices available. The second step is the "option talk", in which the physician lists and explains the options available, with corresponding expected harms and benefits. For some patients, decision aids are a helpful tool in this step, since these tools make options visible and help the patient understand the options in more detail. The third step is the "decision talk", in which the patient's preferences are the most important issue, and the patient and physician together decide what is most preferable.

Joseph-Williams et al. [20] reported various patient-reported barriers and facilitators to shared decision-making, which they divided into two categories: ‘how the healthcare is organized’ and ‘what happens during the decision-making interaction’. Regarding the organization of healthcare, patients reported adequate time for discussion as a facilitator towards shared decision-making. Good continuity of care in terms of, for example, having a personal connection with the care provider, being able to choose which healthcare provider(s) to be involved with, having nurses as “mediator” between a patient and clinician, and optimal coordination between healthcare providers facilitate shared decision-making. Regarding the decision-making interaction, a facilitator is encouraging the active involvement of patients through clearly explaining the value of the patient’s participation in the decision-making process, providing sufficient, understandable information, and enough time to think about the options.

# Supplementary references

1. Simon HA. The Architecture of Complexity. Proc Am Philos Soc. 1962;106(6):467-82.

2. Campagnolo D, Camuffo A. The Concept of Modularity in Management Studies: A Literature Review. Int J Manag Rev. 2010;12(3):259-83. doi: 10.1111/j.1468-2370.2009.00260.x.

3. Bask A, Lipponen M, Rajahonka M, Tinnilä M. Framework for modularity and customization: service perspective. J Bus Ind. 2011;26(5):306-19. doi: 10.1108/08858621111144370.

4. De Blok C, Meijboom B, Luijkx K, Schols J. The human dimension of modular care provision: Opportunities for personalization and customization. Int J Prod Econ. 2013;142(1):16-26. doi: 10.1016/j.ijpe.2012.05.006.

5. Rajahonka M, Bask A, Lipponen M. Modularity and customisation in LSPs' service strategies. Int J Serv Oper Manag. 2013;16:174-204. doi: 10.1504/IJSOM.2013.056165.

6. Fransen L, Peters VJT, Meijboom BR, De Vries E. Modular service provision for heterogeneous patient groups: a single case study in chronic Down syndrome care. BMC Health Serv Res. 2019;19(1):720. doi: 10.1186/s12913-019-4545-8.

7. Peters VJT, Meijboom BR, De Vries E. Interfaces in service modularity: a scoping review. Int J Prod Res. 2018;56(20):6591-606. doi: 10.1080/00207543.2018.1461270.

8. De Blok C, Meijboom B, Luijkx K, Schols J, Schroeder R. Interfaces in service modularity: A typology developed in modular health care provision. J Oper Manag. 2014;32(4):175-89. doi: 10.1016/j.jom.2014.03.001.

9. Voss CA, Hsuan J. Service Architecture and Modularity*. Decis Sci. 2009;40(3):541-69. doi: 10.1111/j.1540-5915.2009.00241.x.

10. Peters V, Vähätalo M, Meijboom B, Barendregt A, Bok LA, Vries E. Elaborating on modular interfaces in multi-provider contexts. Int J Oper Prod Manag. 2020;40:1397-419. doi: 10.1108/IJOPM-12-2019-0822.

11. Soffers R, Meijboom B, Van Zaanen J, Van der Feltz-Cornelis C. Modular health services: a single case study approach to the applicability of modularity to residential mental healthcare. BMC Health Serv Res. 2014;14:210. doi: 10.1186/1472-6963-14-210. PubMed PMID: 24886367.

12. Ekman I, Swedberg K, Taft C, Lindseth A, Norberg A, Brink E, et al. Person-centered care--ready for prime time. Eur J Cardiovasc Nurs. 2011;10(4):248-51. Epub 2011/07/19. doi: 10.1016/j.ejcnurse.2011.06.008. PubMed PMID: 21764386.

13. Barry MJ, Edgman-Levitan S. Shared decision making--pinnacle of patient-centered care. N Engl J Med. 2012;366(9):780-1. Epub 2012/03/02. doi: 10.1056/NEJMp1109283. PubMed PMID: 22375967.

14. Håkansson Eklund J, Holmström IK, Kumlin T, Kaminsky E, Skoglund K, Höglander J, et al. "Same same or different?" A review of reviews of person-centered and patient-centered care. Patient Educ Couns. 2019;102(1):3-11. Epub 2018/09/12. doi: 10.1016/j.pec.2018.08.029. PubMed PMID: 30201221.

15. American Geriatrics Society Expert Panel on Person-Centered Care. Person-Centered Care: A Definition and Essential Elements. J Am Geriatr Soc. 2016;64(1):15-8. Epub 2015/12/03. doi: 10.1111/jgs.13866. PubMed PMID: 26626262.

16. Bastemeijer CM, Voogt L, Van Ewijk JP, Hazelzet JA. What do patient values and preferences mean? A taxonomy based on a systematic review of qualitative papers. Patient Educ Couns. 2017;100(5):871-81. Epub 2017/01/04. doi: 10.1016/j.pec.2016.12.019. PubMed PMID: 28043713.

17. Moore L, Britten N, Lydahl D, Naldemirci Ö, Elam M, Wolf A. Barriers and facilitators to the implementation of person-centred care in different healthcare contexts. Scand J Caring Sci. 2017;31(4):662-73. doi: 10.1111/scs.12376. PubMed PMID: 27859459.

18. Elwyn G, Frosch D, Thomson R, Joseph-Williams N, Lloyd A, Kinnersley P, et al. Shared decision making: a model for clinical practice. J Gen Intern Med. 2012;27(10):1361-7. Epub 2012/05/23. doi: 10.1007/s11606-012-2077-6. PubMed PMID: 22618581.

19. Bomhof-Roordink H, Gärtner FR, Stiggelbout AM, Pieterse AH. Key components of shared decision making models: a systematic review. BMJ Open. 2019;9. doi: 10.1136/bmjopen-2019-031763.

20. Joseph-Williams N, Elwyn G, Edwards A. Knowledge is not power for patients: a systematic review and thematic synthesis of patient-reported barriers and facilitators to shared decision making. Patient Educ Couns. 2014;94(3):291-309. Epub 2013/12/07. doi: 10.1016/j.pec.2013.10.031. PubMed PMID: 24305642.
