# Supplementary material for: How service modularity can provide the flexibility to support person-centered care and shared decision-making
Source: BMC Health Serv Res. 2021 Nov 18;21:1245. doi: 10.1186/s12913-021-07267-6 (PMC8600923; doi:10.1186/s12913-021-07267-6)
Supplement: Supplementary file 2 — Additional file 2. Methodology of scoping review. More detailed explanation of the methodology used for the scoping review. [file 12913_2021_7267_MOESM2_ESM.docx]

# Additional file 2. Methodology of scoping review.

In order to gain insight into the current literature covering service modularity in healthcare, we conducted a scoping review. Scoping reviews are suitable for “comprehensively summarizing and synthesizing evidence to informing practice, programs, and policy and providing direction to future research priorities” [1]. We used the framework published by Arksey and O'Malley [2], further clarified and enhanced by Levac, Colquhoun, and O'Brien [3], to structure the scoping review process. The six stages of this framework are 1. identifying the research question; 2. identifying relevant studies; 3. study selection; 4. charting the data; 5. collating, summarizing, and reporting results; 6. consultation. We used the PRISMA-ScR Checklist for describing the process in a structured manner (S2 Table 1) [4].

## Search strategy

We searched the databases of Elsevier/ScienceDirect, Google Scholar, JSTOR, PubMed, Web of Science & WorldCat Discovery systematically for articles published from 2000 until June 10, 2020. These six databases were selected to cover a wide range of the operations management and healthcare literature. We chose this period since most articles concerning modularity in systems and services are published from 2000 onwards [5]. We used the search strategy **[service] modularity AND health.** The search strategy was adapted to the possibilities of each database (S2 Table 2).

## Study selection

Papers were eligible for inclusion when written in English or Dutch and discussing or applying service modularity in healthcare or when a healthcare setting was studied using a modular frame. After removing the duplicates, 715 unique papers remained for title screening. We excluded papers that did not address service modularity in a healthcare process related to care provision. We screened the articles coming from the searches in three phases. First, two junior researchers (first author [EB] and external researcher [VP]) familiar with service modularity in healthcare screened all titles independently. After screening approximately one hundred titles, these reviewers compared their assessments and discussed their doubts, to make sure they were on the same page before continuing to screen all titles. After screening all titles, the two researchers had an agreement on 95.2 percent of the papers. They discussed their discrepancies and were able to reach agreement for all papers. Simultaneously, one independent senior researcher (second author [BM]) assessed a random selection of 25 percent of all titles. This resulted in 82.8 percent agreement with the final judgments of the junior researchers (EB and VP). The three reviewers discussed the discrepancy in in- or exclusion. In case of doubt or persisting disagreement, articles were included for abstract screening.

Second, one reviewer (EB) screened all abstracts of the remaining 118 papers. Besides, the papers were randomly divided over three senior researchers (second [BM], third [LN], and fourth author [EDV]), whom each reviewed one-third of the abstracts. Comparing these screening results resulted in 78.2 percent agreement with A1. In case of a discrepancy in in- or exclusion, the paper was included for full-text selection.

Third, the reviewers screened the 64 full-texts. Whenever a full-text was not available, the authors tried to retrieve it from their network or contacted the relevant author(s). One junior reviewer (EB) read the full-texts of all remaining papers to determine eligibility. Besides, three senior reviewers (BM, LN, and EDV) each screened one-third of the full-texts. In 87.7 percent of the cases, the reviewers (EB with either BM, LN, or EDV) agreed on the decision to in- or exclude the paper. In case of a discrepancy in inclusion or exclusion, the paper was included for analysis. The full-text screening resulted in the inclusion of 41 papers. However, while coding the papers, we excluded three extra papers that did not provide the information matching our criteria after all or had a great overlap between them. For example, in case a dissertation resulted in a published article with great overlap, we excluded the dissertation and focused on the published article.

One reviewer (EB) scanned the reference lists of the included papers, i.e., ‘snowball method’, to identify additional eligible papers. This resulted in the addition of eleven papers. An independent expert in the field of service modularity in healthcare (MV) reviewed the full set of included papers and checked whether she missed papers. This did not result in any additions. In sum, we included 49 papers for analysis (S2 Table 3).

## Data extraction and analysis

First, we created an overview of basic characteristics (e.g., year and type of paper, context and setting of the research, unit of analysis) of the included papers in Microsoft Excel. Second, we coded the papers in ATLAS.ti (version 8) using a thematic analysis based on the methodology proposed by Gioia et al. [6]. One junior researcher (EB) coded all sections related to service modularity in healthcare within various categories. After coding six papers, we (EB, BM, LN, and EDV) discussed and recognized similarities and differences among the many codes, which we brought back to a manageable number of codes. This set of 1^st^ order concepts was used to code all papers, which was carried out by one researcher (EB). If we stumbled upon a relevant section in a paper for which we did not have a code yet, we created a new code – it was an iterative process. During the coding process, one senior researcher (BM) reviewed five randomly selected papers to assess to what extent he agreed with the coded sections. This resulted in zero to two adjustments per paper. Based on this we decided to proceed with the coding process similarly. After coding all papers, all authors (EB, BM, LN, and EDV) discussed the codes and their content. This resulted in a classification of the 27 codes into six 2^nd^ order themes (S2 Figure 1). Moreover, this discussion resulted in the identification of fifteen promising codes for further analysis. One researcher (EB) created a summary for each of the promising codes, which reflected upon patterns and discrepancies across the included papers within one code. We used this summary to distill the 2^nd^ order themes into so-called “aggregate dimensions” which served as a basis for the Findings section.

## Strengths and limitations

To the best of our knowledge, this is the first paper that explicitly addresses how service modularity can and does serve as a foundation for delivering person-centered care in a shared decision-making context. We conducted a scoping review to comprehensively summarize and synthesize evidence. The research was guided by a research team with expertise in scoping reviews. Moreover, we used a well-established framework [2, 3] to structure the scoping review process and used the PRISMA-ScR Checklist for describing the process [4].

We conducted our search in multiple databases to cover a wide range of the operations management and healthcare literature. Since we used the search strategy [service] modularity AND health, we possibly missed papers that did not use the word “service” in their paper, although covering modularity in a service setting. To deal with this, we used the snowball method and asked an independent expert in the field whether she missed any papers in our final selection.

We included conference papers, published articles, books, and dissertations but did not conduct a critical appraisal of included sources of evidence. We realize not all included papers are fully peer-reviewed (yet). This means we possibly included information from lower quality studies. To minimize this, we relied on the information coming from a published article, if available. Nevertheless, including those different type of papers allowed us to gain a broader perspective on service modularity in healthcare than if we only included peer-reviewed papers.

During the coding and analysis process, we used a thematic analysis based on the methodology proposed by Gioia et al. [6] to guide our process. This helped us to do a thorough analysis of the included papers and extract the relevant information.

# Supplementary references

1. Colquhoun HL, Levac D, O'Brien KK, Straus S, Tricco AC, Perrier L, et al. Scoping reviews: time for clarity in definition, methods, and reporting. J Clin Epidemiol. 2014;67(12):1291-4. Epub 2014/07/19. doi: 10.1016/j.jclinepi.2014.03.013. PubMed PMID: 25034198.

2. Arksey H, O'Malley L. Scoping studies: towards a methodological framework. Int J Soc Res Methodol. 2005;8(1):19-32. doi: 10.1080/1364557032000119616.

3. Levac D, Colquhoun H, O'Brien KK. Scoping studies: advancing the methodology. Implement Sci. 2010;5(1):69. doi: 10.1186/1748-5908-5-69.

4. Tricco AC, Lillie E, Zarin W, O'Brien KK, Colquhoun H, Levac D, et al. PRISMA Extension for Scoping Reviews (PRISMA-ScR): Checklist and ExplanationThe PRISMA-ScR Statement. Ann Intern Med. 2018;169(7):467-73. doi: 10.7326/M18-0850.

5. Campagnolo D, Camuffo A. The Concept of Modularity in Management Studies: A Literature Review. Int J Manag Rev. 2010;12(3):259-83. doi: 10.1111/j.1468-2370.2009.00260.x.

6. Gioia D, Corley K, Hamilton A. Seeking Qualitative Rigor in Inductive Research. Organ Res Methods. 2013;16:15-31. doi: 10.1177/1094428112452151.

7. Peters MD, Godfrey CM, Khalil H, McInerney P, Parker D, Soares CB. Guidance for conducting systematic scoping reviews. Int J Evid Based Healthc. 2015;13(3):141-6. Epub 2015/07/03. doi: 10.1097/xeb.0000000000000050. PubMed PMID: 26134548.

8. Peters MDJ, Godfrey C, McInerney P, Baldini Soares C, Khalil H, Parker D. Chapter 11: Scoping Reviews. In: Aromataris E, Munn Z, editors. Joanna Briggs Institute Reviewer's Manual: JBI; 2017.

# Figures

**
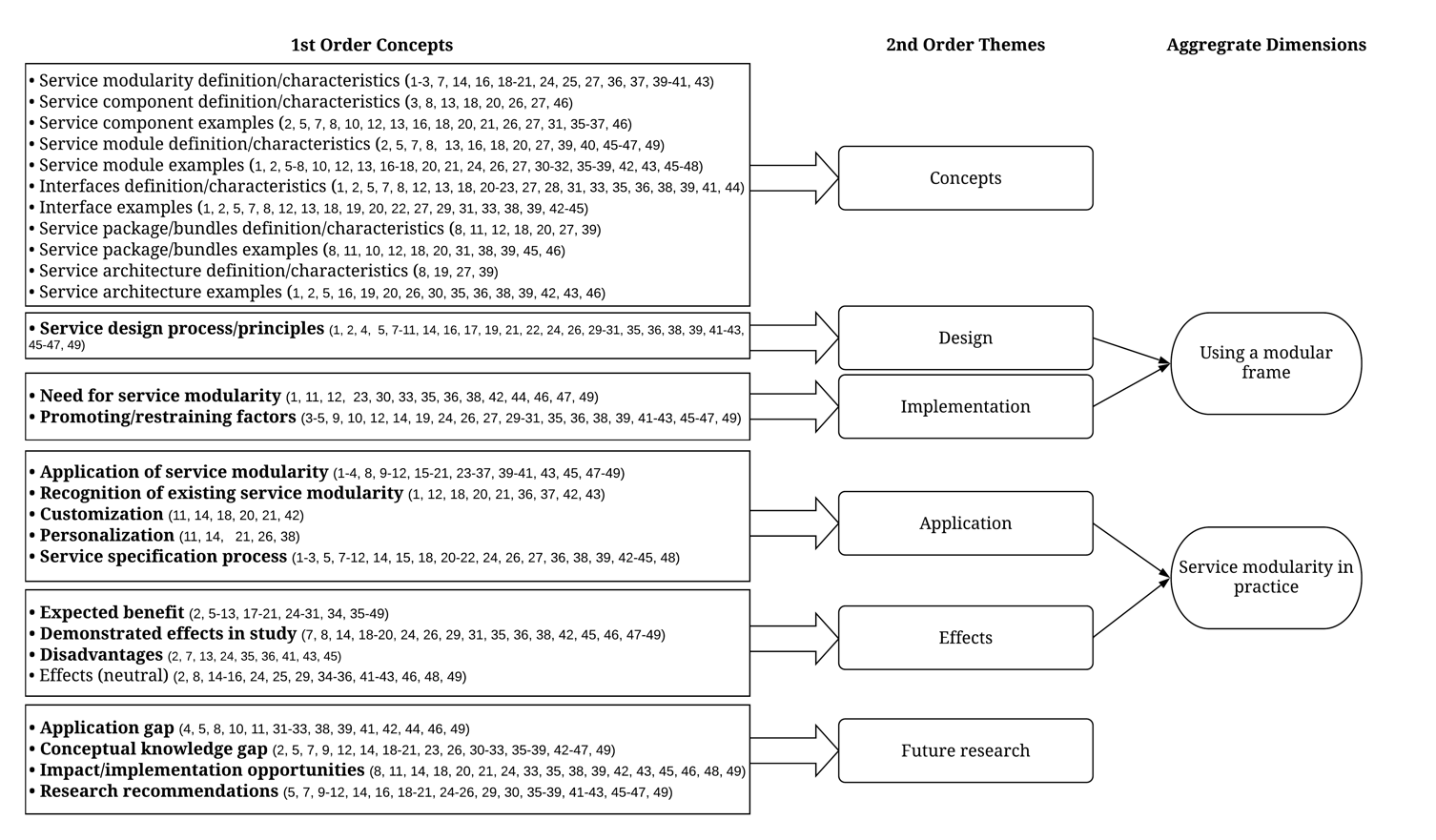
**

**S2 Fig. 1** Data Structure (based on Gioia et al. [6]). Reference numbers correspond with S2 Table 3.

# Tables

| **S2 Table 1. Preferred Reporting Items for Systematic reviews and Meta-Analyses extension for Scoping Reviews (PRISMA-ScR) Checklist [4].** | | | |
| --- | --- | --- | --- |
| Section | Item | Prisma-ScR checklist item | Reported on page # |
| Title | | | |
| Title | 1 | Identify the report as a scoping review. | N/A |
| Abstract | | | |
| Structured summary | 2 | Provide a structured summary that includes (as applicable): background, objectives, eligibility criteria, sources of evidence, charting methods, results, and conclusions that relate to the review questions and objectives. | 1 |
| Introduction | | | |
| Rationale | 3 | Describe the rationale for the review in the context of what is already known. Explain why the review questions/objectives lend themselves to a scoping review approach. | 4, S1 |
| Objectives | 4 | Provide an explicit statement of the questions and objectives being addressed with reference to their key elements (e.g., population or participants, concepts, and context) or other relevant key elements used to conceptualize the review questions and/or objectives. | 4 |
| Methods | | | |
| Protocol and registration | 5 | Indicate whether a review protocol exists; state if and where it can be accessed (e.g., a Web address); and if available, provide registration information, including the registration number. | S1 |
| Eligibility criteria | 6 | Specify characteristics of the sources of evidence used as eligibility criteria (e.g., years considered, language, and publication status), and provide a rationale. | 5, S1-S2 |
| Information sources* | 7 | Describe all information sources in the search (e.g., databases with dates of coverage and contact with authors to identify additional sources), as well as the date the most recent search was executed. | 5, S1 |
| Search | 8 | Present the full electronic search strategy for at least 1 database, including any limits used, such that it could be repeated. | S9 |
| Selection of sources of evidence† | 9 | State the process for selecting sources of evidence (i.e., screening and eligibility) included in the scoping review. | 5, S1-S2 |
| Data charting process‡ | 10 | Describe the methods of charting data from the included sources of evidence (e.g., calibrated forms or forms that have been tested by the team before their use, and whether data charting was done independently or in duplicate) and any processes for obtaining and confirming data from investigators. | 5, S3-S4 |
| Data items | 11 | List and define all variables for which data were sought and any assumptions and simplifications made. | S3, S2 Fig. 1 |
| Critical appraisal of individual sources of evidence§ | 12 | If done, provide a rationale for conducting a critical appraisal of included sources of evidence; describe the methods used and how this information was used in any data synthesis (if appropriate). | N/A |
| Synthesis of results | 13 | Describe the methods of handling and summarizing the data that were charted. | 5, S3-S4 |
| Results | | | |
| Selection of sources of evidence | 14 | Give numbers of sources of evidence screened, assessed for eligibility, and included in the review, with reasons for exclusions at each stage, ideally using a flow diagram. | S2 Fig. 2, S1-S3 |
| Characteristics of sources of evidence | 15 | For each source of evidence, present characteristics for which data were charted and provide the citations. | N/A |
| Critical appraisal within sources of evidence | 16 | If done, present data on critical appraisal of included sources of evidence (see item 12). | N/A |
| Results of individual sources of evidence | 17 | For each included source of evidence, present the relevant data that were charted that relate to the review questions and objectives. | N/A |
| Synthesis of results | 18 | Summarize and/or present the charting results as they relate to the review questions and objectives. | 5-12 |
| Discussion | | | |
| Summary of evidence | 19 | Summarize the main results (including an overview of concepts, themes, and types of evidence available), link to the review questions and objectives, and consider the relevance to key groups. | 12-15 |
| Limitations | 20 | Discuss the limitations of the scoping review process. | S4-S5 |
| Conclusions | 21 | Provide a general interpretation of the results with respect to the review questions and objectives, as well as potential implications and/or next steps. | 12-15 |
| Funding | | | |
| Funding | 22 | Describe sources of funding for the included sources of evidence, as well as sources of funding for the scoping review. Describe the role of the funders of the scoping review. | N/A |

S1 indicates page 1 in Additional file 2.

JBI = Joanna Briggs Institute; PRISMA-ScR = Preferred Reporting Items for Systematic reviews and Meta-Analyses extension for Scoping Reviews.

* Where *sources of evidence* (see second footnote) are compiled from, such as bibliographic databases, social media platforms, and Web sites.

† A more inclusive/heterogeneous term used to account for the different types of evidence or data sources (e.g., quantitative and/or qualitative research, expert opinion, and policy documents) that may be eligible in a scoping review as opposed to only studies. This is not to be confused with *information sources* (see first footnote).

‡ The frameworks by Arksey and O’Malley [2] and Levac and colleagues [3] and the JBI guidance [7, 8] refer to the process of data extraction in a scoping review as data charting*.*

§ The process of systematically examining research evidence to assess its validity, results, and relevance before using it to inform a decision. This term is used for items 12 and 19 instead of "risk of bias" (which is more applicable to systematic reviews of interventions) to include and acknowledge the various sources of evidence that may be used in a scoping review (e.g., quantitative and/or qualitative research, expert opinion, and policy document).

**S2 Table 2. Search Strategy adapted to each Database.**

| Database | Search strategy | Search index |
| --- | --- | --- |
| Elsevier/ScienceDirect | service AND (modular OR modularity) AND (health OR healthcare) | Title, abstract, keywords |
| Google Scholar | “service modularity” AND (health OR healthcare) | Full text |
| JSTOR | (ti:(service*) OR ab:(service*)) AND (ti:(modular*) OR ab:(modular*)) AND (ti:(health*) OR ab:(health*)) | Title and Abstract |
| PubMed | ((service*[Title/Abstract]) AND modular*[Title/Abstract]) AND health*[Title/Abstract] | Title/Abstract |
| Web of Science | (service* AND modular* AND health*) | TOPIC (Title, Abstract, Author, Keywords, Keywords Plus®) |
| Worldcat Discovery | ("service modularity" AND health*) | Keyword |

| **S2 Table 3.** **Included Papers for Analysis.** |
| --- |

1. Barros O. A process architecture pattern and its application to designing health services: emergency case. Bus Process Manag J. 2019;26(2):513-27. doi: 10.1108/BPMJ-08-2018-0210.

2. Blandi V. Customer uncertainty: a source of organizational inefficiency in the light of the modularity theory of the firm [Doctoral Thesis]: University of Trento; 2018.

3. Bohmer RM. Medicine's service challenge: blending custom and standard care. Health Care Manag Rev. 2005;30(4):322-30. Epub 2005/11/18. doi: 10.1097/00004010-200510000-00006. PubMed PMID: 16292009.

4. Brax SA, Bask A, Hsuan J, Voss C. Service modularity and architecture – an overview and research agenda. Int J Oper Prod Manag. 2017;37(6):686-702. doi: 10.1108/IJOPM-03-2017-0191.

5. Broekhuis M, Van Offenbeek M, Eissens-van der Laan M. What professionals consider when designing a modular service architecture? Int J Oper Prod Manag. 2017;37(6):748-70. doi: 10.1108/IJOPM-05-2015-0306.

6. Cardoso S, Aime X, Meininger V, Grabli D, Melo Mora LF, Cohen KB, et al. A Modular Ontology for Modeling Service Provision in a Communication Network for Coordination of Care. Stud Health Technol Inform. 2018;247:890-4. Epub 2018/04/22. PubMed PMID: 29678089.

7. Chorpita BF, Daleiden EL, Weisz JR. Modularity in the design and application of therapeutic interventions. Appl Prev Psychol. 2005;11(3):141-56. doi: https://doi.org/10.1016/j.appsy.2005.05.002.

8. De Blok C, Luijkx K, Meijboom B, Schols J. Improving long-term care provision: towards demand-based care by means of modularity. BMC Health Serv Res. 2010;10:278. PubMed PMID: 20858256.

9. De Blok C, Luijkx K, Meijboom B, Schols J. Modular care and service packages for independently living elderly. Int J Oper Prod Manag. 2010;30(1):75-97. doi: 10.1108/01443571011012389.

10. De Blok C, Meijboom B, Luijkx K, Schols J. Demand-based Provision of Housing, Welfare and Care Services to Elderly Clients: From Policy to Daily Practice Through Operations Management. Health Care Anal. 2009;17(1):68-84. doi: 10.1007/s10728-008-0095-7.

11. De Blok C, Meijboom B, Luijkx K, Schols J. The human dimension of modular care provision: Opportunities for personalization and customization. Int J Prod Econ. 2013;142(1):16-26. doi: 10.1016/j.ijpe.2012.05.006.

12. De Blok C, Meijboom B, Luijkx K, Schols J, Schroeder R. Interfaces in service modularity: A typology developed in modular health care provision. J Oper Manag. 2014;32(4):175-89. doi: 10.1016/j.jom.2014.03.001.

13. De Mattos CS, Fettermann DC, Cauchick-Miguel PA. Service modularity: literature overview of concepts, effects, enablers, and methods. Serv Ind J. 2019:1-22. doi: 10.1080/02642069.2019.1572117.

14. De Pourcq K, Verleye K, Larivière B, Trybou J, Gemmel P. Modularizing Services Based Upon an Actor-Oriented Logic. Acad Manag Proc. 2020;2020(1):18852. doi: 10.5465/AMBPP.2020.18852abstract.

15. Dörbecker R, Böhmann T. The Concept and Effects of Service Modularity – A Literature Review. 2013.

16. Dörbecker R, Tokar O, Böhmann T. Deriving Design Principles for Improving Service Modularization Methods - Lessons Learnt from a Complex Integrated Health Care Service System. 2015.

17. Eissens-Van der Laan M, Broekhuis M, Van Offenbeek M, Ahaus K. Service decomposition: a conceptual analysis of modularizing services. Int J Oper Prod Manag. 2016;36(3):308-31. doi: 10.1108/IJOPM-06-2015-0370.

18. Fransen L, Peters VJT, Meijboom BR, De Vries E. Modular service provision for heterogeneous patient groups: a single case study in chronic Down syndrome care. BMC Health Serv Res. 2019;19(1):720. doi: 10.1186/s12913-019-4545-8.

19. Gittell JH, editor Modularity And The Coordination Of Complex Work. annual conference for Industry Studies; 2008 May 1-2; Boston, MA.

20. Gobbi C, Hsuan J. Modularity in Cancer Care Provision. In: Van Donk PD, De Koster R, De Leeuw S, Fransoo J, Van der Veen J, editors. Proceedings of the 4th World Conference of Production & Operations Management. Brussels: EUROMA, European Operations Management Association; 2012.

21. Silander K, Särkilahti A, Torkki P, Peltokorpi A, Tarkkanen M, Kaila M. Perspectives of mass customisation and modularisation in health service delivery: a scoping review. Int J Healthc Technol Manag. 2019;17:255. doi: 10.1504/IJHTM.2019.10026709.

22. Kapsali M, Bessant J. Improving Patient Safety - How to Use Service Modularity in Healthcare Processes to Manage Systemic Errors. POMS conference; 11/06; Reno, Nevada, USA.2012.

23. Lubarski A. Understanding service modularity - antecedents, processes, and operationalization [Doctoral Thesis]: Universität Bremen FB7 Wirtschaftswissenschaften; 2019.

24. Lyon AR, Lau AS, McCauley E, Stoep AV, Chorpita BF. A case for modular design: Implications for implementing evidence-based interventions with culturally-diverse youth. Prof Psychol Res Pr. 2014;45(1):57-66. doi: 10.1037/a0035301. PubMed PMID: 25328279.

25. Lyon AR, Ludwig K, Romano E, Koltracht J, Vander Stoep A, McCauley E. Using modular psychotherapy in school mental health: provider perspectives on intervention-setting fit. J Clin Child Adolesc Psychol. 2014;43(6):890-901. doi: 10.1080/15374416.2013.843460. PubMed PMID: 24134063.

26. Meijboom B, Vaessen P, Van der Heijden K, van Sambeeck MFAC, Gemmel P. Customisation and personalization in clinical pathways using a modular perspective. Proceedings of the 25th European Operations Management Association (EurOMA 2018). Budapest2018.

27. Meijboom BR, Van den Bosch L, Schalk R. Refining case management for dementia using insights from operations management. Quality in Ageing and Older Adults. 2014;15(3):162-70. doi: 10.1108/QAOA-04-2014-0005.

28. Meyer M, Jekowsky E, Crane F. Applying platform design to improve the integration of patient services across the continuum of care. Manag Serv Qual. 2007;17:23-40. doi: 10.1108/09604520710720656.

29. Micheli GJL, Trucco P, Sabri Y, Mancini M. Modularization as a system life cycle management strategy: Drivers, barriers, mechanisms and impacts. Int J Eng Bus Manag. 2019;11:184797901882504. doi: 10.1177/1847979018825041.

30. Peters C, Leimeister JM. TM3-A modularization method for telemedical services: design and evaluation. Proceedings of 21st European …. 2013. PubMed PMID: rayyan-4333676.

31. Peters V, Barendregt A, Meijboom B, Bok LA, De Vries E. Enhancing coordination in complex modular hospital care provision. 25th International Annual European Operations Management Association. Budapest2018.

32. Peters V, Meijboom B, Bunt JE, De Winter P, Bok V, Van Steenbergen M, et al., editors. Examining modular service architecture for complex healthcare: a means to more person-centered healthcare? 10th Service Operations Management Forum (SOMF) and the 8th International Seminar on Service Modularity; 2019 2019/1.

33. Peters VJT, Meijboom BR, De Vries E. Interfaces in service modularity: a scoping review. Int J Prod Res. 2018;56(20):6591-606. doi: 10.1080/00207543.2018.1461270.

34. Schuler B, Lee B, Kolivoski K, Attman N, Lindsey M. Implementing a Modular Research-Supported Treatment in Child Welfare: Effects and Obstacles. Res Soc Work Prac. 2014;26. doi: 10.1177/1049731514563988.

35. Silander K, Torkki P, Lillrank P, Peltokorpi A, Brax SA, Kaila M. Modularizing specialized hospital services: Constraining characteristics, enabling activities and outcomes. Int J Oper Prod Manag. 2017;37(6):791-818. doi: 10.1108/IJOPM-06-2015-0365.

36. Silander K, Torkki P, Peltokorpi A, Lepäntalo A, Tarkkanen M, Bono P, et al. Modularising outpatient care delivery: A mixed methods case study at a Finnish University Hospital. Health Serv Manag Res. 2018;31(4):195-204. doi: 10.1177/0951484817752629. PubMed PMID: 29336174.

37. Silander K, Torkki P, Peltokorpi A, Tarkkanen M, Lepäntalo A, Mattson J, et al. Comparing modular and personal service delivery in specialised outpatient care: A survey of haematology and oncology patient preferences. Health Serv Manag Res. 2019;32(4):209-17. Epub 2019/08/14. doi: 10.1177/0951484819868681. PubMed PMID: 31403337.

38. Soffers R, Meijboom B, Hsuan J, editors. Principles for Implementing Modularity in Healthcare. 23rd EurOMA Conference; 2016; Trondheim, Norway.

39. Soffers R, Meijboom B, Van Zaanen J, Van der Feltz-Cornelis C. Modular health services: a single case study approach to the applicability of modularity to residential mental healthcare. BMC Health Serv Res. 2014;14:210. doi: 10.1186/1472-6963-14-210. PubMed PMID: 24886367.

40. Spring M, Santos J. Interfaces in Service and Process Modularity. 5th International Seminar on Service Architecture and Modularity; January 16-17; Copenhagen2014.

41. Srivastava S, Prakash G. Enhancing Modularity in Healthcare Services Through Integration. Asia-Pac J Manag Res Innov. 2019;15(3):97-110. doi: 10.1177/2319510X19883077.

42. Vähätalo M. Modularity in Health and Social Services: A Systematic Review. Int J Pub Priv Healthc Manag. 2012;2(1):21-jul. PubMed PMID: rayyan-4335467.

43. Vähätalo M, Kallio T. Organising health services through modularity. Int J Oper Prod Manag. 2015;35:925-45. doi: 10.1108/IJOPM-12-2013-0523.

44. Vähätalo M, Peters V, Meijboom B, Barendregt A, Bok V, De Vries E, editors. (Cross) organizational interfaces supporting integration of health and social services. 10th Service Operations Management Forum (SOMF) and the 8th International Seminar on Service Modularity; 2019 2019/1.

45. Van Brunt DL. Modular cognitive-behavioral therapy: Dismantling validated treatment programs into self-standing treatment plan objectives. Cogn Behav Pract. 2000;7(2):156-65. doi: https://doi.org/10.1016/S1077-7229(00)80026-7.

46. Van der Laan MR. The feasibility of modularity in professional service design: Towards low cost person-centred care [Doctoral Thesis]. Groningen: University of Groningen; 2015.

47. Villatte JL, Vilardaga R, Villatte M, Plumb Vilardaga JC, Atkins DC, Hayes SC. Acceptance and Commitment Therapy modules: Differential impact on treatment processes and outcomes. Behav Res Ther. 2016;77:52-61. PubMed PMID: 26716932.

48. Weisz JR, Chorpita BF, Palinkas LA, Schoenwald SK, Miranda J, Bearman SK, et al. Testing standard and modular designs for psychotherapy treating depression, anxiety, and conduct problems in youth: a randomized effectiveness trial. Arch Gen Psychiatry. 2011;69(3):274-82. Epub 2011/11/09. doi: 10.1001/archgenpsychiatry.2011.147. PubMed PMID: 22065252.

49. Zhang X, Ma S, Chen S. Healthcare process modularization using design structure matrix. Adv Eng Inform. 2019;39:320-30. doi: 10.1016/j.aei.2019.02.005. PubMed PMID: rayyan-4333582.
